# Efficacy and Safety of Chuan Huang Fang Combining Reduced Glutathione in Treating Acute Kidney Injury (Grades 1–2) on Chronic Kidney Disease (Stages 2–4): Study Protocol for a Multicenter Randomized Controlled Clinical Trial

**DOI:** 10.1155/2022/1099642

**Published:** 2022-03-15

**Authors:** Ling Chen, Xuezhong Gong

**Affiliations:** Department of Nephrology, Shanghai Municipal Hospital of Traditional Chinese Medicine, Shanghai University of Traditional Chinese Medicine, 274 Zhijiang Middle Road, Shanghai 200071, China

## Abstract

**Background:**

Acute kidney injury (AKI) is a global public health challenge resulting in considerable morbidity and mortality. AKI on chronic kidney disease (CKD) (AKI on CKD, A on C) accounts for about a third of total AKI. For severe AKI grade 3, renal replacement therapy (RRT) should be implemented in time. However, the lack of recognized drug treatment method for AKI grades 1–2 is a crucial problem in clinic. Chuan Huang Fang (CHF) is a Chinese herbal formulation developed for the treatment of A on C from the Shanghai Municipal Hospital of Traditional Chinese Medicine. Our previous studies suggested that CHF might effectively protect renal functions of A on C patients. As a widely used antioxidant in clinic, reduced glutathione (RG) is reported to improve the clinical efficacy of high-flux hemodialysis (HFHD) in severe AKI patients recently. To address the crucial problem mentioned above, thus we design a new clinical protocol of CHF combining RG and try to evaluate the efficacy and safety of this protocol in treating patients diagnosed with CKD stages 2–4 complicated with AKI grades 1–2.

**Methods:**

This is a multicenter randomized controlled clinical trial. We intend to enroll 162 participants, and these participants will be divided into the RG group, the CHF group, and the RG + CHF group randomly assigning 1 : 1 : 1 principle. The RG group will be general treatments combining RG, the CHF group will be general treatments combining CHF, and the RG + CHF group will be general treatments combining RG and CHF. The duration of treatment will last two weeks. The primary evaluation outcome will be the change in the slope of serum creatinine (Scr) over 2 weeks. Secondary evaluation outcomes include changes in blood urea nitrogen (BUN), estimated glomerular filtration rate (eGFR), urinary AKI biomarkers (neutrophil gelatinase-associated lipocalin (NGAL), interleukin-18 (IL-18), gamma-glutamyl transpeptidase (*γ*-GT), etc.), traditional Chinese medicine (TCM) symptoms, inflammatory indicators, and oxidative stress indicators. Meanwhile, the vital sign indicators and adverse events (AEs) will be closely observed. These dates will be meticulously recorded and properly handled by investigators throughout the study. *Discussion*. This study will provide convictive research-derived data to evaluate clinical efficacy and safety of CHF combing RG for CKD stages 2–4 complicated with AKI grades 1–2 and provide an evidence-based recommendation for clinicians. The timely completion of this trial will provide a novel drug treatment method for A on C. This trial is registered with ChiCTR2100043311 and registered on February 9, 2021.

## 1. Introduction

AKI is defined as an abrupt decrease in kidney function, which encompasses both structural damage and functional loss [1]. According to 2012 Kidney Disease Improving Global Outcomes (KDIGO-2012) AKI definition [2], it is diagnosed by an increase in Scr to at least 0.3 mg/dl (26.5 *μ*mol/l) within 48 hours, or an increase in Scr to more than 1.5 times baseline during prior 7 days, or a urine volume decrease to less than 0.5 ml/kg/h for 6 hours. AKI is a global public health challenge [3], it is a severe complication caused by various critical diseases leading to considerable morbidity and mortality both in short term and long term [4, 5], and it implicates a great burden in sanitary costs [6]. A on C accounts for about a third of total AKI [7]. It is estimated that there are more than 13 million patients with AKI worldwide every year [8]. US statistical data indicate that the incidence of AKI is about 2100 per million [9]. A study on the clinical status of AKI in China points out that over 2 million patients are hospitalized with AKI, and about 700,000 patients died of AKI every year [10].

For severe AKI (grade 3), RRT should be implemented as soon as possible. It is reported that about 26.9% of patients with AKI require RRT eventually [11]. Current modalities of RRT for AKI include intermittent hemodialysis (IHD), continuous renal replacement therapy (CRRT), and peritoneal dialysis (PD). Also, sustained low-efficiency dialysis (SLED) has been put forward as an alternative RRT method and applied to clinical practice in many medical institutions worldwide [2, 12]. Recently, although AKI has attracted more and more attention and lots of related basic or clinical studies have been accomplished, there is still a lack of recognized drug treatment method for AKI grades 1–2 [13–15].

Studies have confirmed that oxidative stress injuries exist in the occurrence of AKI, and it plays an important role in affecting the prognosis of AKI [16]. At the same time, oxidative stress might induce inflammation by activating macrophages and complement systems, which would aggravate the potential kidney damage [16, 17]. Therefore, it is of great significance to pay more attention to the effect of oxidative stress in AKI.

Furthermore, several studies have shown that increasing pro-inflammatory biomarkers in AKI patients have a close connection with an elevated short-term mortality [18, 19]. Studies on AKI show that tumor necrosis factor-*α* (TNF-*α*), interleukin-6 (IL-6), and other cytokines have a significant meaning in the process of inflammation mediation [20]. During the early period of AKI, renal blood flow insufficiency might lead to sub-fatal renal tubular injury, and the inflammatory mediators generated by the injury might lead to vasoconstriction and aggravate ischemia. In addition, A on C may be the result of combined effects of abnormal hemodynamics, inflammation, oxidative stress, decreased prostaglandin synthesis, and increased synthesis of thromboxane in renal cortex [15, 21–23].

Based on the published data, during RRT unpopular period, Chinese doctors used to treat AKI with TCM methods [24]. CHF is a Chinese herbal formulation of Professor Gong Xuezhong for treating A on C developed from the Shanghai Municipal Hospital of Traditional Chinese Medicine [25], and our previous small sample clinical studies suggest that CHF might effectively protect renal functions of A on C patients by preventing oxidant damage and inhibiting inflammation [15, 26, 27]. In the process of AKI, numerous oxygen-free radicals are generated, endogenous antioxidants are constantly consumed, and large numbers of inflammatory factors are released, aggravating kidney injury [28]. Reduced glutathione (RG) can effectively control the local inflammatory response, reduce the accumulation of reactive oxygen species in renal tissues, lower the level of inflammatory factors, and relieve oxidative stress in organs and tissues [29–31]. Recently, RG is reported to improve the clinical efficacy of HFHD in patients with severe AKI [32, 33]. Therefore, we plan to further optimize the original protocol, using CHF combining RG to form a new treatment method and finally carry out such a multicenter randomized controlled clinical trial on CKD stages 2–4 complicated with AKI grades 1–2 to evaluate the clinical efficacy and safety of this new method. The timely completion of this trial will provide a novel drug treatment method for A on C.

## 2. Methods

### 2.1. Objectives

This clinical trial is based on years of research studies in the early stage, which lays a solid foundation for successful completion. The aim was to evaluate the efficacy and safety of new treatment method CHF combining RG in treating A on C.

### 2.2. Study Design and Setting

This is a multicenter randomized controlled clinical trial in which participants will be recruited from the department of nephrology. Three hospitals are listed below: (1) Shanghai Municipal Hospital of Traditional Chinese Medicine; (2) Shuguang Hospital Affiliated to Shanghai University of Traditional Chinese Medicine; and (3) Minhang Branch of Yueyang Hospital of Integrative Chinese and Western Medicine Affiliated to Shanghai University of Traditional Chinese Medicine.

The design of this trial follows strict scientific clinical research methodology and complies with principles of *the Declaration of Helsinki* [34] and *the Guidelines for Good Clinical Practice* [35].

**Figure 1:**
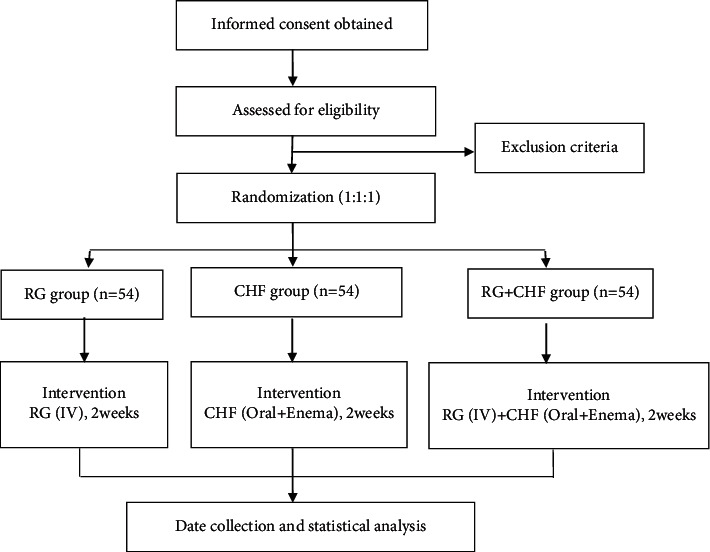
Flow chart of the study. RG: reduced glutathione, CHF: Chuan Huang Fang.

This trial is registered in the Chinese Clinical Trial Register (No. ChiCTR2100043311). The study flow chart is shown in Figure 1.

### 2.3. Participants

This trial plans to enroll potential eligible participants diagnosed with CKD stages 2–4 complicated with AKI grades 1–2 with spleen-kidney qi deficiency and toxicity stasis inter-combination syndrome. To encourage patients to participate in the trial, we will post recruitment posters in the above three hospitals and trial advertisements will also be accessed through study's Web page. Besides, we will pay attention to enrollment progress to make sure it could reach the target sample size.

#### 2.3.1. Diagnostic Criteria for AKI

According to KDIGO clinical practice guideline for acute kidney injury [2] and Quality Control Manual of Shanghai Nephrology Department [36], patients would be diagnosed with AKI if (1) increase in Scr by ≥ 0.3 mg/dl (≥26.5 *μ*mol/l) within 48 hours; or (2) increase in Scr to ≥1.5 times baseline during prior 7 days; or (3) urine volume <0.5 ml/kg/h for 6 hours. If patients cannot provide Scr baseline, clinicians can refer to Scr value within the last 3 months (no more than 1 year at the longest). If the value is still not available, patients should be required to repeat testing Scr within 24 hours to help diagnose AKI. KDIGO classification of AKI is as follows (Table 1).

#### 2.3.2. Diagnostic Criteria for CKD

CKD is characterized by structural or functional abnormalities in the kidney, and the complex symptom situation lasts for more than 3 months, impacting health well-being [37]. KDIGO classification of CKD is as follows (Table 2).

#### 2.3.3. Diagnostic Criteria for TCM Syndrome Differentiation

According to Guidelines for Clinical Research of Chinese Medicine (New Drug) [38] and Diagnosis, Syndrome Differentiation, and Efficacy Evaluation of Chronic Renal Failure (Trial Protocol) [39], patients conformed to 4 primary symptoms and 1–2 secondary symptoms listed below can be diagnosed with spleen-kidney qi deficiency and toxicity stasis inter-combination syndrome. The primary symptoms are as follows: (1) fatigue or shortness of breath and reluctance to speak; (2) nausea and vomiting; (3) dim complexion; and (4) soreness in waists and knees. The secondary symptoms are as follows: (1) abdominal fullness and distention; (2) lack of appetite and numb to react; (3) squamous and dry skin; (4) thick and greasy tongue coating; (5) dark and purple tongue or petechiae; and (6) fine tart or slow sunken pulse.

#### 2.3.4. Inclusion Criteria

Participants will be enrolled in the trial if they satisfy all the following criteria: (1) meet the diagnostic criteria for CKD stages 2–4 and AKI grades 1–2; (2) meet the diagnostic criteria of TCM syndrome differentiation; (3) 24 h U-pro ≤2.5 g; (4) between 18 and 70 years old; and (5) voluntary to be enrolled in the clinical trial and sign informed consents.

#### 2.3.5. Exclusion Criteria

Participants will not be enrolled in the trial if they have any of the following criteria: (1) pregnancy or lactation; (2) with serious primary diseases of other organs in urgent need of immediate treatment or with malignant tumors, active tuberculosis, and other consumption diseases; (3) with anorectal diseases not suitable for enema; (4) kidney transplantation; (5) psychopaths, patients who cannot cooperate; (6) allergic to therapeutic medicine; and (7) participating in clinical trials or participated in other clinical trials within 3 months.

#### 2.3.6. Withdrawal Criteria

Participants will withdraw from the trial if (1) poor subject compliance; (2) allergic to therapeutic medicine; (3) cases lost to follow-up in the trial; (4) patients request to withdraw from the trial; (5) cannot meet the inclusion criteria after inclusion; and (6) serious diseases or complications occur suddenly during the trial that has no causal relationship with AKI. Participants developing serious diseases or complications during the treatment cannot be withdrawn until the assessment of causal relationship with AKI has been accomplished. Complications such as cardiorenal syndrome and sepsis usually indicate the progression of AKI and should not be simply excluded from the efficacy analysis. Reasons should be given for withdrawal cases, and related forms should be reserved for future reference. Those cases are not statistically analyzed for efficacy, but adverse reactions should be analyzed for those who have received at least one treatment and can be determined to have records of adverse reactions. Participants who withdraw within two weeks after treatment will be defined as missing case and will not be enrolled in the pee-protocol population.

#### 2.3.7. Shedding Criteria

Natural shedding and loss of follow-up during observation may occur during the trial. He/she will quit by himself/herself if it is not suitable to continue participating in the trial because of severe complications or unpredictable events. Shedding cases should be documented in detail, and intention-to-treat (ITT) analysis should be performed in the end.

#### 2.3.8. Trial Suspension Criteria

It is essential to suspend the trial if serious safety problems occur during the process of trial. The chief supervisor will be in charge of dealing with this particular situation when it occurs. Critical issues should be recorded in detail and handled properly. All collected data will only be analyzed in the end, and no planned interim analyses will be performed.

### 2.4. Randomization and Blinding

A simple random method is adopted, and SPSS (version 21.0) software will be used by independent biostatisticians to generate random number sequences. Written informed consent should be signed immediately by himself/herself, agreeing to voluntarily enroll in the trial. Participants should be consented for randomization and participation. According to the sequence of enrolling in clinical observation, participants will be randomly numbered and divided into the RG group, the CHF group, and the RG + CHF group by investigators at 1 : 1 : 1 ratio. During randomization producer, sealed envelopes marked with sequential coding numbers will be adopted to keep allocation details, and group allocation should be kept blind to patients, biostatisticians, and investigators. As placebo is not used in the control group, it is not possible to be blind in the treatment. However, it is masked from staff performing all laboratory investigations.

### 2.5. Interventions

Patients diagnosed with CKD stages 2–4 complicated with AKI grades 1–2 will be enrolled after signing written informed consents. They will be randomly divided into the RG group, the CHF group, and the RG + CHF group. To minimize potential loss and help participants conduct the trial smoothly, three group patients will receive study behavior and health education, and they will also be told possible risks and benefits, their rights and obligations, and how to handle an emergency situation. All patients will receive a low-salt, high-quality, and low-protein diet (protein intake 0.6–0.8 g/kg/d). Also, they will receive general treatments, whose purposes are to correct water, electrolyte, and acid-base balance disorders, control blood pressure, improve anemia, and correct renal bone diseases. Designed investigations will keep in touch with participants regularly and assist them to receive treatment and testing on schedule.

The RG group will be given RG 1.8 g added to 0.9% normal saline or 5% glucose 250 ml for injection, intravenous injection (IV), once per day for 2 weeks. The CHF group will be given CHF orally, twice per day for 2 weeks. At the same time, the concentrated solution of CHF will be given an enema, once per day for 5 days, and then rest for 2 days, 5 times in a week. The RG + CHF group will be the combination of the RG group and the CHF group, and intervention methods are the same as the above.

Pharmaceutical compositions of CHF are mainly as follows: prepared rhubarb (Zhida Huang), Ligusticum wallichii (Chuan Xiong), Codonopsis pilosula (Dangshen), Coptidis rhizome (Huanglian), Smilacis glabrae (Tufuling), etc. RG will be produced by Chongqing Yaoyou Pharmaceutical Co. Ltd., and CHF will be produced by Jiangyin Tianjiang Pharmaceutical Co. Ltd. Therefore, the quantity and quality of medicine can be effectively guaranteed.

Related examinations will be conducted and collected at baseline, every 1 week during treatment. Patient information will be recorded, and efficacy-related examinations, mechanism-related indicators, safety-related indicators, and drug management will be closely observed. Measured items and time points are listed in Table 3.

### 2.6. Outcome Measures

The primary evaluation outcome will be the change in the slope of Scr over 2 weeks. Posttreatment changes in BUN, eGFR, and urinary AKI biomarkers including urinary NGAL, IL-18, *γ*-GT, and TCM symptoms will be the secondary evaluation outcomes.

In addition to the secondary evaluation outcome, inflammatory indicators including TNF-*α* and IL-6 and oxidative stress indicators including heme oxygenase-1 (HO-1), malondialdehyde (MDA), and superoxide dismutase (SOD) will also be measured before and after treatment.

Safety outcomes and vital sign indicators (electrocardiogram, blood routine, urine routine, serum potassium, hemoglobin, etc.) will be recorded once before and 2 weeks after medication. Meanwhile, AEs will be closely observed, meticulously recorded, and properly handled by investigators throughout the study.

HCG: human chorionic gonadotropin; Scr: serum creatinine; BUN: blood urea nitrogen; eGFR: estimated glomerular filtration rate; TCM: traditional Chinese medicine; NGAL: neutrophil gelatinase-associated lipocalin: IL-18: interleukin-18; *γ*-GT: gamma-glutamyl transpeptidase; IL-6: interleukin-6; TNF-*α*: tumor necrosis factor-*α*; HO-1: heme oxygenase-1; MDA: malondialdehyde; SOD: superoxide dismutase.

### 2.7. Evaluation Criteria for TCM Syndrome Effect

Statistics are carried out according to the improvement of clinical symptom score with reference to the *Guidelines for Clinical Research of Chinese Medicine (New Drug)* [38], and the primary and secondary symptoms of spleen-kidney qi deficiency and toxicity stasis inter-combination syndrome will be classified as mild, moderate, and severe with 4, 8, and 12 points and 2, 4, and 8 points, respectively. The two total scores calculated by the measurement scale according to TCM symptoms for each patient will be summed up, and then, they will be used to calculate efficacy indicator (EI):

  EI = (total symptom score before treatment -total symptom score after treatment)/total symptom score before treatment *X* 100%
  EI is adopted to evaluate the treatment efficacy after treatment. Symptom improvement levels will be demonstrated in following categories: clinical control (EI ≥ 95%), significant effect (94% > EI ≥ 70%), and effectiveness (69% > EI ≥ 30%) to inefficacy (EI < 30%).

### 2.8. Data Entry and Management

Case report forms (CRFs) will be filled out by investigators, and each selected participant must complete CRFs. All information in CRFs should be accurately and truthfully recorded. Completed CRFs will be inspected by the clinical supervisor, and then, they should be delivered to the data administrators. Data entry and management will be accomplished by designated data administrators. Patients' personal information should be kept strictly confidential. All date should be recognized through coding numbers without displaying their personal information directly. All date will not be accessible without the permission of the supervisor. The data administrator will use EpiData software to compile data entry software and carry out data entry and management. The supervisor will be in charge of data entry and management procedure. As GCP requires, all date is required to keep for at least five years in the database after the trial accomplishes.

### 2.9. Sample Size Estimation

The sample size will be calculated according to the primary outcome of baseline Scr change. Based on previous studies and literature reports [26, 30], the mean value of Scr was 160.39 *μ*mol/l, and the standard deviation was 44.43 *μ*mol/l. We thus hypothesize that Scr for CHF combing RG group would be significantly lower than the other two groups by more than 30 *μ*mol/l. It is calculated using the sample size estimation software PASS (version 15.0.3) to achieve test efficacy 1–*β* = 0.90 and significant difference *α* = 0.05 (one-sided test). Considering 20% drop rate, the estimated sample size for randomization will be 54 cases in each group for a total of 162 cases.

### 2.10. Statistical Analysis

The principal analysis will be conducted on a full analysis set (FAS), which consists of participants randomly allocated into two groups. Participants completing and complying well with the study protocol without major violation will be referred to the per-protocol population. All participants taking test drugs will be enrolled in safety analysis and conform with the ITT principle.

For non-normally distributed variables, mean ± standard deviation or median will be brought to describe continuous variables. Categorical variables will be presented in the form of numbers and percentages. The difference in Scr will be tested using the one-way ANOVA with repeated measures. The secondary outcomes will be compared between two groups using the independent-samples Student's *t*-tests for normally distributed continuous data and using Pearson's chi-square test (or Fisher's exact test if cell count <5 in any cell) for categorical data. Two-sided tests will be used for all statistical tests, and *P* < 0.05 will be considered statistically significant. SPSS (version 21.0) software will be used to carry out statistical analysis by a biostatistician not involved in the study. No additional analyses or interim analysis will be performed.

### 2.11. Quality Control of Data

Before initiating the trial, a conference will be held in Shanghai to make sure all participants can receive centralized training. They will receive professional training to ensure accurate, complete, and timely recording of observations and results in the CRFs. The clinical protocol is designed on the foundation of expertise and clinical experience of all participants to ensure it is scientific and operational. Patients will be selected strictly according to inclusion and exclusion criteria, and the entire trial process will follow a multicenter randomized controlled clinical design. At each site, test sample materials including blood, urine, and other materials will be collected and delivered to the central laboratory for analysis and destruction to reduce data bias. If there are AEs during the trial, appropriate treatments should be taken for subjects and it is necessary to report the situation to authorities immediately. Biostatisticians will participate in designing the trial and analyzing collected data up to the end of the trial. Data administrators will primarily conduct manual checks of data entry and then perform systematic checks after completion. The data monitoring committee (DMC) will meet regularly to monitor the conduct of the trial and evaluate quality control of data.

### 2.12. Safety Assessment

In this trial, AEs are recognized as negative or untoward medical manifestations during the study conducting period, regardless of the possibility of a causal relationship. CHF has been applied to clinical treatments and animal experiments for many years and proved an effective prescription, and no obvious AEs have been observed. According to the package insert of RG, it may have skin rash and other allergic symptoms such as anorexia, nausea, upper abdominal discomfort, and other gastrointestinal symptoms occasionally. Relevant AEs occurring in this trial will be recorded systematically in detail throughout the trial. Meticulous description of event cause, subsequent interventions, handling outcome, and other relevant information will also be included in the form. If AEs occur, the patient will be closely observed and properly taken care of until AEs disappear. It is essential to carry out follow-up investigations. Besides, the results should be recorded in detail and then reasons will be analyzed. Serious adverse events (SAEs) are as follows: (1) critical or life-threatening complications; (2) hospitalization or disability, even death; and (3) other serious event significant hazards to participants. AEs and SAEs will be monitored and evaluated by researchers, and then, researchers will give related participants proper treatments and report them to authorities. They will also be reported in trial publications at the end of the study.

### 2.13. Ethics Approval

This trial must be conducted in accordance with *the Declaration of Helsinki* [34], and it has been approved by the international review board of each participating hospital. This trial has been approved by the Ethics Committee of the Shanghai Municipal Hospital of Traditional Chinese Medicine (No. 2020SHL-KYYS-60) and other ethics committees at each center. When each patient is enrolled, investigators should introduce the purpose, procedure, and possible risks of the study and then ask patients to sign informed consent forms. The relevant personal information of participants and potential participants will be kept confidential throughout the whole trial process and will not be disclosed to anyone else other than staff in charge of the trial.

## 3. Discussion

Considering AKI is a clinical syndrome defined as rapid loss of renal function and CKD is characterized by structural or functional abnormalities in the kidney, AKI and CKD are closely connected. Meanwhile, AKI is a common factor causing CKD. While AKI-to-CKD transition has already been numerously reported, studies of A on C are relatively limited [3–6]. It is well known that AKI occurring in patients with CKD is commonly more intractable to handle. Besides, CKD is also a significant hazardous element for the progress of AKI [40]. Related studies demonstrated that the preexistence of CKD jeopardized renal function in AKI patients and postponed its recovery after AKI [41].

TCM has been widely used in the treatment of kidney diseases in China [42]. CHF is a Chinese herbal formulation for treating A on C and has achieved positive clinical efficacy in our previous clinical studies [15, 26, 27]. Tetramethylpyrazine (TMP), as an active ingredient of CHF and herbal medicine Ligusticum wallichii (Chuanxiong), could prevent AKI by the possible mechanisms of alleviating oxidative stress injury, inhibiting inflammation, preventing apoptosis of intrinsic renal cells, and regulating autophagy [43–45]. Recently, increasing clinical and experimental studies suggest that TMP might effectively prevent AKI in several AKI models [43, 46, 47].

According to the KDIGO guidelines, the main treatment strategies for AKI are to stabilize the body's internal environment and most treatments are supportive [2]. During the early stage of AKI, it is essential to find out the major cause, maintain hemodynamic stabilization, and deal with complex complications [48]. In consideration of autoregulation mechanisms damaged in AKI, maintaining hemodynamic stabilization should be attached great significance to [49]. As stated above, RG could clear away the harmful peroxide metabolites, restrain oxidative stress, and play certain metabolic roles in inflammatory factors [29–31].

RRT is intended for severe AKI grade 3, while there still lacks recognized drug treatment method for AKI grades 1–2 [11, 13–15]. Based on our previous clinical data and the current developments of A on C clinical treatment, we thus design such a new protocol about the drug treatment method of CHF combining RG for CKD stages 2–4 complicated with AKI grades 1–2. We hope that the present multicenter randomized controlled clinical trial will be helpful to solve this clinical problem. It could be considered that CHF combining RG might effectively reduce renal injury in patients with A on C, promote recovery of renal function, and improve clinical symptoms of patients, and thus it has acceptable clinical application values. On this basis, we will further observe the curative effect of combination CHF with RG for A on C patients to obtain more direct clinical evidence.

## Figures and Tables

**Table 1:** KDIGO classification of AKI.

Stage	Serum creatinine	Urine output
1	1.5–1.9 times baseline, or ≥0.3 mg/dl (26.5 *μ*mol/l) increase	<0.5 ml/kg/h for 6–12 h
2	2.0–2.9 times baseline	<0.5 ml/kg/h for≥12 h
3	3.0 times baseline, or≥4.0 mg/dl (353.6 *μ*mol/l), or initiation of RRT, or < 18 years, eGFR <35 ml/min per1.73 m^2^	<0.3 ml/kg/h for≥24 h or anuria for ≥12 hours

**Table 2:** KDIGO classification of CKD.

GFR category	GFR (ml/min/1.73 m^2^)	Terms
**G1**	≥90	Normal or high
**G2**	60–89	Mildly decreased
**G3a**	45–59	Mildly to moderately decreased
**G3b**	30–44	Moderately to severely decreased
**G4**	15–29	Severely decreased
**G5**	<15	Kidney failure

**Table 3:** SPIRIT table: measured items and time points.

Items	Baseline	Treatment	
	0 week	1 week	2 weeks
General	×		
**Informed consent**	×		
**Inclusion and exclusion criteria**	×		
**General information**	×		
**General physical examination**	×		
**Urine HCG (only women)**	×		
**Medical and drug use history**	×		
**Concomitant disease and treatment**	×		
**Combined medication**	×	×	×
Randomization	×		
Intervention			
**RG group**		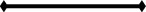	
**CHF group**		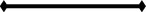	
**RG** **+** **CHF group**		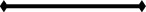	
Effectiveness observation			
**Renal functions (Scr, BUN, eGFR)**	×		×
**AKI biomarkers (NGAL, IL-18, *γ*-GT)**	×		×
**TCM symptoms**	×		×
Mechanism observation			
**Inflammatory indicators (IL-6, TNF-*α*)**	×		×
**Oxidative stress indicators (HO-1, MDA, SOD)**	×		×
Safety observation			
**Electrocardiogram**	×		×
**Blood routine**	×		×
**Urine routine**	×		×
**Fecal routine and occult blood test**	×		×
**Serum potassium**	×		×
**Hemoglobin**	×		×
**Adverse events**		×	×
Drug management			
**Drug distribution**	×	×	×
**Drug recycling**		×	×
**Drug count**		×	×
Research conclusion			×

## Data Availability

It is not applicable for the submitted manuscript to share primary data as databases have not been established or analyzed during this present period. Further information unaddressed would be accessible from the corresponding author after the trial is completed.

## Abbreviations

AKI: Acute kidney injury
CKD: Chronic kidney disease
RRT: Renal replacement therapy
CHF: Chuan Huang Fang
RG: Reduced glutathione
HFHD: High-flux hemodialysis
Scr: Serum creatinine
BUN: Blood urea nitrogen
eGFR: Estimated glomerular filtration rate
NGAL: Neutrophil gelatinase-associated lipocalin
IL-18: Interleukin-18
*γ-*GT: Gamma-glutamyl transpeptidase
TCM: Traditional Chinese medicine
AEs: Adverse events
IHD: Intermittent hemodialysis
CRRT: Continuous renal replacement therapy
PD: Peritoneal dialysis
SLED: Sustained low-efficiency dialysis
TNF-*α*: Tumor necrosis factor-*α*
IL-6: Interleukin-6
ITT: Intention-to-treat
IV: Intravenous injection
HO-1: Heme oxygenase-1
MDA: Malondialdehyde
SOD: Superoxide dismutase
HCG: Human chorionic gonadotropin
EI: Efficacy indicator
CRFs: Case report forms
FAS: Full analysis set
DMC: Data monitoring committee
SAEs: Serious adverse events.

## Supplementary Materials

Supplementary Materials S1: ethical approval document. S2: SPIRIT 2013 Checklist. S3: copy of the original funding document. S4: original version of the informed consent document.
