# Supplementary material for: Efficacy and Safety of Chuan Huang Fang Combining Reduced Glutathione in Treating Acute Kidney Injury (Grades 1–2) on Chronic Kidney Disease (Stages 2–4): Study Protocol for a Multicenter Randomized Controlled Clinical Trial
Source: Evid Based Complement Alternat Med. 2022 Mar 15;2022:1099642. doi: 10.1155/2022/1099642 (PMC8941542; doi:10.1155/2022/1099642)
Supplement: Supplementary Materials — S1: ethical approval document. S2: SPIRIT 2013 Checklist. S3: copy of the original funding document. S4: original version of the informed consent document. [file 1099642.f1.zip › 1099642.f1/S1 Ethical approval document(Original).pdf]

上海市中医医院伦理委员会
临床科研项目伦理审查意见

意见号：2020SHL-KYYS-60

项目名称	川黄方联合还原型谷胱甘肽方案治疗2-4期CKD合并1-2级AKI的多中心随机对照临床研究		
任务来源	上海市科学技术委员会	课题编号	
研究负责单位	上海市中医医院		
本中心主要研究者（姓名及职称）	龚学忠 主任医师		
审查日期	2020年08月05日	审查地点	上海市芷江中路274号
审查类别	初始审查	审查方式	快速审查
审查文件	伦理审查申请表<br>项目申报书		
审查要点	研究项目是否符合基本伦理原则 ■是 □否<br>方案设计科学合理，具有可行性 ■是 □否		
审查意见	根据卫生部《涉及人的生物医学研究伦理审查办法》（2016）、世界医学会《赫尔辛基宣言》、以及国际医学科学组织委员会颁布的《人体生物医学研究国际道德指南》的伦理原则，伦理委员会对本科研项目申报进行了审查，意见如下：<br>同意龚学忠的“川黄方联合还原型谷胱甘肽方案治疗2-4期CKD合并1-2级AKI的多中心随机对照临床研究”申报各级科研项目。当项目批准立项后，研究者必须将正式研究方案及知情同意书等提交本伦理委员会审查，获得批准后方可实施。		
审查结果	■ 同意申报 修改后同意申报 □不同意申报		
盖　章	上海市中医医院伦理委员会		
日　期	2020年8月5日		
