# Supplementary material for: Efficacy and Safety of Chuan Huang Fang Combining Reduced Glutathione in Treating Acute Kidney Injury (Grades 1–2) on Chronic Kidney Disease (Stages 2–4): Study Protocol for a Multicenter Randomized Controlled Clinical Trial
Source: Evid Based Complement Alternat Med. 2022 Mar 15;2022:1099642. doi: 10.1155/2022/1099642 (PMC8941542; doi:10.1155/2022/1099642)
Supplement: Supplementary Materials — S1: ethical approval document. S2: SPIRIT 2013 Checklist. S3: copy of the original funding document. S4: original version of the informed consent document. [file 1099642.f1.zip › 1099642.f1/S1 Ethical approval document(Translation).pdf]

Shanghai Municipal Hospital of Traditional Chinese Medicine Ethics Committee

Application Form for Ethical Review of Clinical Research Projects

Identifier: 2020SHL-KYYS-60

Project Name	Chuanhuang Fang combining reduced glutathione in treating acute kidney injury (grades 1-2) on chronic kidney disease (stages 2-4): study protocol for a multicenter randomized controlled clinical trial		
Project Sources	Shanghai Science and Technology Commission	Identifier	
Research Institution in Charge	Shanghai Municipal Hospital of Traditional Chinese Medicine		
Responsible Person	Xuezhong Gong, Chief Physician		
Review Date	August 5, 2020	Review Site	No.274, Zhijiang Middle Road, Shanghai
Review Category	Initial review	Review Method	Quick Review
Review Documents	1. Application form for ethical review<br>2. Project declaration		
Review Key Points	1.Research projects are consistent with basic ethical principles<br>■ Agree □ Disagree<br>2. The protocol design is reasonable and feasible<br>■ Agree □ Disagree		
Review Comments	According to the *Methods for the Ethical Review of Biomedical Research Involving Human Subjects (2016)* issued by the National Health and Family Planning Commission, *Declaration of Helsinki* issued by World Medical Association (WMA), *International Ethical Guidelines for Biomedical Research Involving Human Subjects* issued by Council International Organization of Medical Sciences (CIOMS), The ethics committee reviewed the report of this scientific research project, opinions are as follows:<br>Agree with professor xuezhong gong’s project *Chuanhuang Fang combining reduced glutathione in treating acute kidney injury (grades 1-2) on chronic kidney disease (stages 2-4): study protocol for a multicenter randomized controlled clinical trial* to apply for scientific research projects at all levels. After the project is approved, the investigator must submit the		

	formal study protocol and informed consent to the ethics committee for review. It can be implemented only after approval is obtained.
Review Result	■ Agree<br>□ Agree after necessary modifications<br>□ Disagree
Seal	
Date of Review Completion	August 5, 2020
