# Supplementary material for: Efficacy and Safety of Chuan Huang Fang Combining Reduced Glutathione in Treating Acute Kidney Injury (Grades 1–2) on Chronic Kidney Disease (Stages 2–4): Study Protocol for a Multicenter Randomized Controlled Clinical Trial
Source: Evid Based Complement Alternat Med. 2022 Mar 15;2022:1099642. doi: 10.1155/2022/1099642 (PMC8941542; doi:10.1155/2022/1099642)
Supplement: Supplementary Materials — S1: ethical approval document. S2: SPIRIT 2013 Checklist. S3: copy of the original funding document. S4: original version of the informed consent document. [file 1099642.f1.zip › 1099642.f1/S3 Funding documentation of the NSFC (No.81873280 )(Original) (1).pdf]

关于国家自然科学基金资助项目批准及有关事项的通知

龚学忠　先生/女士：

根据《国家自然科学基金条例》的规定和专家评审意见，国家自然科学基金委员会（以下简称自然科学基金委）决定批准资助您的申请项目。项目批准号：81873280，项目名称：从BNIP3和PINK1/Parkin介导的线粒体自噬和线粒体质量控制研究对比剂急性肾损伤的发病机制及“制大黄-川芎”药对的肾保护机制，直接费用：62.00万元，项目起止年月：2019年01月至 2022年 12月，有关项目的评审意见及修改意见附后。

请尽早登录科学基金网络信息系统（https://isisn.nsfc.gov.cn），获取《国家自然科学基金资助项目计划书》（以下简称计划书）并按要求填写。对于有修改意见的项目，请按修改意见及时调整计划书相关内容；如对修改意见有异议，须在计划书电子版报送截止日期前提出。

计划书电子版通过科学基金网络信息系统（https://isisn.nsfc.gov.cn）上传，由依托单位审核后提交至自然科学基金委进行审核。审核未通过者，返回修改后再行提交；审核通过者，打印为计划书纸质版（一式两份，双面打印），由依托单位审核并加盖单位公章后报送至自然科学基金委项目材料接收工作组。计划书电子版和纸质版内容应当保证一致。向自然科学基金委提交和报送计划书截止时间节点如下：

1、提交计划书电子版截止时间为**2018年9月11日16点**（视为计划书正式提交时间）；

2、提交计划书电子修改版截止时间为**2018年9月18日16点**；

3、报送计划书纸质版截止时间为**2018年9月26日16点**。

请按照以上规定及时提交计划书电子版，并报送计划书纸质版，未说明理由且逾期不报计划书者，视为自动放弃接受资助。

附件：项目评审意见及修改意见表

国家自然科学基金委员会
医学科学部
2018年8月16日
