# Supplementary material for: Efficacy and Safety of Chuan Huang Fang Combining Reduced Glutathione in Treating Acute Kidney Injury (Grades 1–2) on Chronic Kidney Disease (Stages 2–4): Study Protocol for a Multicenter Randomized Controlled Clinical Trial
Source: Evid Based Complement Alternat Med. 2022 Mar 15;2022:1099642. doi: 10.1155/2022/1099642 (PMC8941542; doi:10.1155/2022/1099642)
Supplement: Supplementary Materials — S1: ethical approval document. S2: SPIRIT 2013 Checklist. S3: copy of the original funding document. S4: original version of the informed consent document. [file 1099642.f1.zip › 1099642.f1/S3 Funding documentation of the NSFC (No.82074387 )(Original) (1).pdf]

国家自然科学基金资助项目批准通知

龚学忠　先生/女士：

根据《国家自然科学基金条例》规定和专家评审意见，国家自然科学基金委员会（以下简称自然科学基金委）决定资助您申请的项目。项目批准号：82074387，项目名称：从GPX4、p62/Nrf2/HO-1介导的肾小管上皮细胞铁死亡研究三价砷致肾损伤的发病机制及川黄方的肾保护机制，直接费用：55.00万元，项目起止年月：2021年01月至 2024年 12月，有关项目的评审意见及修改意见附后。

请尽早登录科学基金网络信息系统（https://isisn.nsfc.gov.cn），获取《国家自然科学基金资助项目计划书》（以下简称计划书）并按要求填写。对于有修改意见的项目，请按修改意见及时调整计划书相关内容；如对修改意见有异议，须在电子版计划书报送截止日期前向相关科学处提出。

电子版计划书通过科学基金网络信息系统（https://isisn.nsfc.gov.cn）上传，依托单位审核后提交至自然科学基金委进行审核。审核未通过者，返回修改后再行提交；审核通过者，打印纸质版计划书（一式两份，双面打印），依托单位审核并加盖单位公章，将申请书纸质签字盖章页订在其中一份计划书之后，一并将上述材料报送至自然科学基金委项目材料接收工作组。电子版和纸质版计划书内容应当保证一致。**自然科学基金委将对申请书纸质签字盖章页进行审核，对存在问题的，允许依托单位进行一次修改或补齐。**

向自然科学基金委补交申请书纸质签字盖章页、提交和报送计划书截止时间节点如下：

1. **2020年10月23日16点：**提交电子版计划书的截止时间（视为计划书正式提交时间）；

2. **2020年10月30日16点：**提交电子修改版计划书的截止时间；

3. **2020年11月06日16点：**报送纸质版计划书（其中一份包含申请书纸质签字盖章页）的截止时间。

4. **2020年11月27日16点：**报送修改后的申请书纸质签字盖章页的截止时间。

请按照以上规定及时提交电子版计划书，并报送纸质版计划书和申请书纸质签字盖章页，未说明理由且逾期不报计划书或申请书纸质签字盖章页者，视为自动放弃接受资助；未按要求修改或逾期提交申请书纸质签字盖章页者，将视情况给予暂缓拨付经费等处理。

附件：项目评审意见及修改意见表

国家自然科学基金委员会

2020年9月27日
