# Supplementary material for: Efficacy and Safety of Chuan Huang Fang Combining Reduced Glutathione in Treating Acute Kidney Injury (Grades 1–2) on Chronic Kidney Disease (Stages 2–4): Study Protocol for a Multicenter Randomized Controlled Clinical Trial
Source: Evid Based Complement Alternat Med. 2022 Mar 15;2022:1099642. doi: 10.1155/2022/1099642 (PMC8941542; doi:10.1155/2022/1099642)
Supplement: Supplementary Materials — S1: ethical approval document. S2: SPIRIT 2013 Checklist. S3: copy of the original funding document. S4: original version of the informed consent document. [file 1099642.f1.zip › 1099642.f1/S3 Funding documentation of the NSFC (No.82074387 )(Translation).pdf]

Notice on the approval and related matters of the project funded by the National Natural Science Foundation of China

Dear Mr Xuezhong Gong:

In accordance with the *Regulations of the National Natural Science Foundation of China (NSFC)* and the opinions of experts, NSFC decides to fund the project you have applied for. Project approval identifier: No.82074387. Project name: Study on the pathogenesis of trivalent arsenic induced kidney injury and the renal protective mechanisms of Chuanhuang Fang from GPX4, p62/Nrf2/HO-1-mediated ferroptosis of renal tubular epithelial cells. Direct costs: 550000 Yuan. Project duration: January 2021 to December 2024. The modification and review comments of the project are attached.

Please visit the Internet-based Science Information System (https://isisn.nsfc.gov.cn) as soon as possible to obtain the NSFC Project Proposal and fill in as required. For the projects with modification suggestions, please adjust the relevant contents of the plan in time according to the modification suggestions. Any objection to the modification suggestions should be raised to the relevant office before the deadline for submission of the electronic version of the project plan.

The electronic version of the project plan should be uploaded to the Internet-based Science Information System (https://isisn.nsfc.gov.cn). After verification by the supporting institution, it shall be submitted to NSFC for further examination. Those who fail to pass the examination shall be submitted after revising. The applicant who has passed the examination shall print the plan (in two copies, printed on both sides), which shall be examined and stamped by the supporting institution. And then the signature and seal page of the printed application form shall be affixed to one of the printed documents. Finally, the above documents shall be submitted to the Project Materials Receiving Working Group of NSFC. NSFC will review the signature and seal page of the printed application. If there is any problem, the supporting institution is allowed once to revise or submit it.

The deadline for resubmitting the signature and seal page of the printed application form, uploading and submitting the project plan to NSFC:

1. **16:00 on October 23, 2020:** The deadline for uploading the electronic version of the project plan (regarded as the official submission time);

2. **16:00 on October 30, 2020:** The deadline for uploading the electronic revised project plan;

3. **16:00 on November 6, 2020:** The deadline for submitting the printed project plan (one of which contains the signature and seal page of the printed application form);

4. **16:00 on November 27, 2020:** The deadline for submitting the signature and seal page of the printed revised application form.

Please uploading the electronic version of the project plan in time according to the above provisions, and submit the printed version and the signature and seal page of the printed application form. Those who fail to submit the plan or the signature and seal page of the printed application form before the deadline without giving reasons will be deemed to automatically give up accepting the funding. If the applicant fails to revise the application form as required or fails to submit the signature and seal page of the application form in time, he/she will be postponed the appropriation of funds according to the circumstances.

Attachment: Project Review Comments and Modification Suggestions Table

National Natural Science Foundation

September 27, 2020
