# Supplementary material for: Efficacy and Safety of Chuan Huang Fang Combining Reduced Glutathione in Treating Acute Kidney Injury (Grades 1–2) on Chronic Kidney Disease (Stages 2–4): Study Protocol for a Multicenter Randomized Controlled Clinical Trial
Source: Evid Based Complement Alternat Med. 2022 Mar 15;2022:1099642. doi: 10.1155/2022/1099642 (PMC8941542; doi:10.1155/2022/1099642)
Supplement: Supplementary Materials — S1: ethical approval document. S2: SPIRIT 2013 Checklist. S3: copy of the original funding document. S4: original version of the informed consent document. [file 1099642.f1.zip › 1099642.f1/S3 Funding documentation of the STCSM (No.20Y21902200 )(Original).pdf]

基金项目资助、科研计划立项证明

兹证明我单位 龚学忠 同志参与承担了下列基金项目、科研课题的研究。

项目批准单位：上海市科学技术委员会

基 金 名 称：上海市“科技创新行动计划”医学创新研究专项项目

立项课题名称：川黄方联合还原型谷胱甘肽治疗 2-4 期 CKD 合并 1-2 级 AKI 的多中心随机、对照临床研究

项 目 编 号：20Y21902200

项 目 成 员：龚学忠

立 项 时 间：2020 年 11 月 17 日

项目完成时间：2020 年 12 月至 2023 年 11 月

特此证明。

上海中医药大学附属市中医医院 科研处

单位名称（盖章）：

时　　间：2021.7.22
