# Supplementary material for: Efficacy and Safety of Chuan Huang Fang Combining Reduced Glutathione in Treating Acute Kidney Injury (Grades 1–2) on Chronic Kidney Disease (Stages 2–4): Study Protocol for a Multicenter Randomized Controlled Clinical Trial
Source: Evid Based Complement Alternat Med. 2022 Mar 15;2022:1099642. doi: 10.1155/2022/1099642 (PMC8941542; doi:10.1155/2022/1099642)
Supplement: Supplementary Materials — S1: ethical approval document. S2: SPIRIT 2013 Checklist. S3: copy of the original funding document. S4: original version of the informed consent document. [file 1099642.f1.zip › 1099642.f1/S3 Funding documentation of the STCSM (No.20Y21902200 )(Translation).pdf]

基金项目资助、科研计划立项证明

Certificate of project support and scientific research plan approval

兹证明我单位龚学忠同志参与承担了下列基金项目、科研课题的研究。

This file is to certify that Professor Xuezhong Gong of our department has participated in the research of the following fund projects and scientific research.

项目批准单位:上海市科学技术委员会

Project approval unit: Shanghai Science and Technology Committee (STCSM)

基金名称:上海市"科技创新行动计划"医学创新研究专项项目

Fund Name: Medical innovation research special project of Shanghai Scientific and Technological Innovation Action Plan

立项课题名称:川黄方联合还原型谷胱甘肽治疗 2-4 期 CKD 合并 1-2 级 AKI 的多中心随机、对照临床研究

Project name: Chuanhuang Fang combining reduced glutathione in treating acute kidney injury (grades 1-2) on chronic kidney disease (stages 2-4): study protocol for a multicenter randomized controlled clinical trial

项目编号: 20Y21902200

Project Number: 20Y21902200

项目成员:龚学忠

Project member: Xuezhong Gong

立项时间: 2020 年 11 月 17 日

Project approval time: November 17, 2020

项目完成时间:2020 年 12 月至 2023 年 11 月

Project completion time: December 2020 to November 2023

时间: 2021.7.22

Date: July 22,20021
