# Supplementary material for: Efficacy and Safety of Chuan Huang Fang Combining Reduced Glutathione in Treating Acute Kidney Injury (Grades 1–2) on Chronic Kidney Disease (Stages 2–4): Study Protocol for a Multicenter Randomized Controlled Clinical Trial
Source: Evid Based Complement Alternat Med. 2022 Mar 15;2022:1099642. doi: 10.1155/2022/1099642 (PMC8941542; doi:10.1155/2022/1099642)
Supplement: Supplementary Materials — S1: ethical approval document. S2: SPIRIT 2013 Checklist. S3: copy of the original funding document. S4: original version of the informed consent document. [file 1099642.f1.zip › 1099642.f1/S4 Original version of the informed consent document (Original).pdf]

知情同意书•知情告知页

尊敬的受试者：

我们将邀请您参加一项针对 A on C 的临床研究，进一步评价川黄方联合还原型谷胱甘肽方案治疗 A on C 的疗效与安全性。

在您决定是否参加这项研究之前，请尽可能仔细阅读以下内容，它可以帮助您了解该项研究以及为什么要进行这项研究，研究的程序和期限。以及参加研究后可能给您带来的益处、风险和不适。以下是本项研究的介绍：

一、研究背景和研究目的

本研究拟在前期小样本临床试验和多个动物实验都证实有效及基础上，进一步优化原有方案，以川黄方(口服+灌肠)联合还原型谷胱甘肽组成新的中西医结合治疗方案，开展对 2-4 期 CKD 合并 1-2 级 AKI 患者的多中心、随机、对照临床研究，以期获得川黄方联合还原型谷胱甘肽有效治疗 A on C 的循证医学证据，并阐明急性肾损伤标志物在早期诊断 A on C 中的作用，及 CKD 微炎症状态和炎症小体在 A on C 中扮演的角色，最终分别为难治性疾病 A on C 的早期诊断、临床治疗提供敏感性检测指标和有效的中西医结合治疗方法，并有助于阐明本病的发病机制。

本研究将在上海市中医医院肾内科、上海中医药大学附属曙光医院肾内科、上海中医药大学附属岳阳医院闵行分院(上海市闵行区中西医结合医院)肾内科 3 个中心进行，预计有 162 名受试者自愿参加。

本项研究已经过伦理委员会评议，认为此项研究是遵循赫尔辛基宣言，符合医学伦理道德的。

二、入选标准和排除标准

2.1 入选标准

①符合 CKD2-4 期的诊断标准和急性肾损伤 1-2 级的诊断标准；
②符合脾肾亏虚、毒瘀互结证的中医证候诊断标准；
③24h U-pro≤2.5 克；
④年龄在 18 岁-70 岁；
⑤患者自愿参加本次临床实验，并签署知情同意书者。

2.2 排除标准

①妊娠或准备妊娠及哺乳期妇女；
②合并有其他脏器严重原发性疾病急需立即治疗，或有恶性肿瘤、活动性结核等消耗系统疾病；
③患有肛门直肠疾病不适宜灌肠治疗
④肾移植术后；

⑤精神病患者、不能合作者；
⑥对治疗药物过敏者；
⑦正在参加其它药物临床试验者或 3 个月内参加过其他临床试验者。

三、如果参加研究需要做什么？

在入选本项研究前，您将接受以下检查以确定您是否可以参加研究：1.医生将询问、记录您的病史，对您进行体格检查。2.您需要做血常规、尿常规、肾功能等理化检查。筛选后，如果您符合入选条件，您接受何种治疗药物方案将根据计算机产生的随机药物编码表决定，您将有可能入选还原型谷胱甘肽组、川黄方组或者还原型谷胱甘肽联合川黄方组。

需要您配合的其他事项：

请您按医生和您约定的随访时间来医院就诊，您的随访对于本项研究非常重要，因为医生将判断您接受的治疗是否真正起作用。

四、可能从本项研究中获得的收益。此种收益包括：

1.您的病情有可能获得改善；

2.您将在研究期间获得良好的医疗服务；

3.部分药物或理化检查可能减免。

五、参加研究可能的不良反应、风险和不便

所有治疗药物都有可能产生副作用。如果在研究中您出现任何不适，或病情发生新的变化，或任何意外情况，不管是否与药物有关，均应及时通知您的医生，医生将对此做出判断和医疗处理。

医生将尽全力预防由于本研究可能带来的伤害。如果在临床试验中出现不良事件，医学专家委员会将会鉴定不良事件是否与试验药物有关。申办者将对试验相关的损害提供治疗的费用及相应的经济补偿，这一点已经在我国《药品临床试验质量管理规范》中做出了规定。

您在研究期间需要按时到医院随访，这些都可能给您造成麻烦或带来不便。

六、您个人信息的保密

您的医疗记录（包括研究病历及理化检查报告、CRF）将按规定保存在医院。研究者、申办者代表、伦理委员会和药品监督管理部门将被允许查阅您的医疗记录。任何有关本项研究结果的公开报告将不会披露您的个人身份。我们将在允许范围内，尽一切努力保护您个人医疗资料的隐私。

除本研究以外，有可能在今后的其他研究中会再次利用您的医疗记录。您现在也可以声明拒绝除本研究外的其他研究利用您的医疗记录。

七、您可以获得更多的信息

您可以在任何时间提出有关本项研究的任何问题。您的医生将给您留下医院和自己的电话号码以便能回答您的问题。

如果在研究过程中有任何重要的新信息，可能影响您继续参加研究的意愿时，您的医生将会及时通知您。

八、可以自愿选择参加研究和中途退出研究，是否参加本项研究完全取决于您的自愿。您可以拒绝参加此项研究，或在研究过程中的任何时间退出研究，这不会影响您和医生的关系，也不会影响对您的医疗或有其他方面利益的损失。

您的医生或研究者出于对您的最大利益考虑，可能会随时中止您参加本项研究。

如果您不参加本项研究，或中途退出，还有很多其它替代的治疗药物。您不必为了治疗疾病而必须选择参加本项研究。

如果您因为任何原因从研究中退出，您可能被询问有关您使用试验药物情况。如果医生认为需要，您也可能被要求进行实验室检查和体格检查。

如果您选择参加本项研究，我们希望您能够坚持完成全部研究过程。

九、是否参加本项研究由您自己决定。您可以和您家人或者亲友讨论后再做出决定。在您做出参加研究的决定前，请尽可能向您的医生询问有关问题，直至您对本项研究完全理解。

感谢您阅读以上材料。如果您决定参加本项研究，请告诉您的医生，医生会为您安排一切有关研究的事务。

请您保留这份资料。

知情同意书•同意签字页

同意声明：

1.我已阅读了有关本研究的介绍，而且有机会就此研究与医生讨论并提出问题。

2.我知道参加本研究的受益和风险。我知道参加研究是自愿的，我确认已有充足的时间对此进行考虑，而且明白：

（1）初次就诊时详细向医生讲述自己的病史，并且进行相应的检查和化验，以便医生做出准确的判断，决定我是否接受此研究。

（2）如果符合条件，我将接受系统的治疗；并且在治疗期间，我需要做相应的检查和化验；如果因病情变化我需要采取其他药物治疗，我会事先征求医生意见，或者事后如实告诉医生。

（3）我同样清楚，如果我中途退出研究，特别是由于药物的原因使我退出研究时，我需将病情变化告诉医生，完成相应的体检和理化检查，这将对我本人和整个研究十分有利。

（4）我可随时了解与试验有关信息资料，在试验的任何阶段都有权随时退出试验而不会遭到歧视或报复，我在退出此研究后，将不会影响我接受其它有效的治疗。

（5）我同意药品监督管理部门，伦理委员会或申办者代表团查阅我的研究资料。

（6）我同意本研究以外的其他研究利用我的医疗记录。

我已明白上述情况，并决定参加此临床研究，并书面同意。

受试者（或代理人）签名：______________

联系电话：______________

签名日期：______年_____月______日

我确认已向患者解释了本试验的详细情况，包括其权利以及可能的获益和风险。

研究者签名：_______________

联系电话：______________

签名日期：______年_____月______日
