# Supplementary material for: Efficacy and Safety of Chuan Huang Fang Combining Reduced Glutathione in Treating Acute Kidney Injury (Grades 1–2) on Chronic Kidney Disease (Stages 2–4): Study Protocol for a Multicenter Randomized Controlled Clinical Trial
Source: Evid Based Complement Alternat Med. 2022 Mar 15;2022:1099642. doi: 10.1155/2022/1099642 (PMC8941542; doi:10.1155/2022/1099642)
Supplement: Supplementary Materials — S1: ethical approval document. S2: SPIRIT 2013 Checklist. S3: copy of the original funding document. S4: original version of the informed consent document. [file 1099642.f1.zip › 1099642.f1/S4 Original version of the informed consent document (Translation).pdf]

Informed Consent • Informed Notification Page

Dear participants:

We would like to invite you to participate in a clinical study of acute kidney injury on chronic kidney disease (AKI on CKD, A on C) to further evaluate the efficacy and safety of Chuanhuang Fang (CHF) combining reduced glutathione (RG) method in the treatment of A on C.

Before you decide to participate in the study, please read the following introduction as carefully as possible to help you understand the whole study and why it is being conducted, as well as procedure and duration of the study. And the benefits, possible risks and discomforts you may experience if you participate in the study. The following is an introduction to this study:

1. Background and purpose

This study intends to further optimize the original protocol based on the effectiveness of small sample clinical trials and multiple animal experiments during the early stage. We thus design a new clinical protocol of CHF (oral + enema) combining RG and try to evaluate the efficacy and safety of this protocol in treating patients diagnosed with CKD stages 2-4 complicated with AKI grades 1-2. The current study will provide convictive research-derived data to evaluate clinical efficacy and safety of CHF combing RG for A on C patients, and provide an evidence-based recommendation for clinicians. This study will clarify the biomarkers of AKI in the early diagnosis of A on C, and the role of microinflammation in A on C patients. Finally, sensitive biomarkers and effective integrated traditional Chinese medicine and Western medicine treatment methods will be provided for the early diagnosis and clinical treatment of refractory A on C, and the pathogenesis of this disease will be elucidated.

This is a multicenter randomized controlled clinical trial in which participants will be recruited from department of nephrology. Three hospitals are listed below: (1) Shanghai Municipal Hospital of Traditional Chinese Medicine ;(2) Shuguang Hospital Affiliated to Shanghai University of Traditional Chinese Medicine;(3) Minhang Branch of Yueyang Hospital of Integrative Chinese & Western Medicine

Affiliated to Shanghai University of Traditional Chinese Medicine. It is estimated 162 participants would be enrolled in the study.

The study was reviewed by the Ethics Committee as medically ethical in accordance with the *Declaration of Helsinki.*

2.Inclusion criteria and exclusion criteria

2.1 Inclusion criteria

Participants will be enrolled in the trial if they satisfy all the following criteria: (1) meet the diagnostic criteria for CKD stages 2-4 and AKI grades 1-2; (2) meet the diagnostic criteria of TCM syndrome differentiation; (3) 24 h U- pro ≤ 2.5g; (4) between 18 and 70 years old; (5) voluntary to be enrolled in clinical trial and sign informed consents.

2.2 Exclusion criteria

Participants will not be enrolled in the trial if they have any of the following criteria: (1) pregnancy or lactation; (2) with serious primary diseases of other organs in urgent need of immediate treatment or with malignant tumors, active tuberculosis and other consumption diseases; (3) with anorectal diseases not suitable for enema; (4) kidney transplantation; (5) psychopaths, patients who can’t cooperate; (6) allergic to therapeutic medicine; (7) participating in clinical trials or participated in other clinical trials within 3 months.

3.What should you do if you participate in the study?

Before being enrolled in the study, you will undergo the following tests to determine whether you are eligible to participate in the study: (1) Investigators will ask you about your medical history and give you a physical examination. (2) You need to take blood routine, urine routine, kidney function test and other necessary tests. After screening, if you are eligible for inclusion, according to the sequence of enrolling in clinical observation, participants will be randomly numbered and divided into RG group, CHF group and RG+CHF group by investigators at 1:1:1 ratio.

Other matters requiring your cooperation:

Please come to the hospital according to the follow-up time agreed by the investigators. Your follow-up is very important for this study, because the investigators will judge whether the treatment you received really works.

4. Possible benefits you may benefit from this research. Such benefits include:

(1) Your physical condition is likely to improve;

(2) You will receive good medical services during the study period;

(3) Some drugs, physical and chemical tests may be exempted.

5. Possible adverse reactions, risks and inconveniences

All therapeutic drugs may have side effects. If you experience any discomfort during the study, or any unexpected physical conditions, whether related to drugs or not, you should inform investigators in a timely manner, and investigators will make judgments and medical treatments.

Investigators will do their best to prevent any harm that may result from this study. If an adverse event occurs during the clinical trial, a committee of medical experts will determine whether the event is related to the investigative drugs. The sponsors will provide treatment costs and corresponding economic compensation for trial-related damage, which has been stipulated in the *Quality Management Standard for Drug Clinical Trials in China*.

You need to visit the hospital on time during the study, which may cause trouble or inconvenience to you.

6. Confidentiality of your personal information

Your medical records (including research records, physical and chemical examination reports and CRFs) will be kept in the hospital according to regulations. Researchers, sponsor representatives, ethics committees, and drug regulatory authorities will be allowed access to your medical records. Your personal identity will not be disclosed in any public reports based on the results of this study. We will do everything we can to protect privacy of your personal medical information.

In addition to this study, it is possible that your medical records will be reused in future studies. You may also declare that you refuse to use your medical records for studies other than this one.

7. You can get more information

You may ask any questions about this study at any time. Investigators will give you the hospital's phone number and his/her own telephone number so he/she can answer your questions.

If there are any significant new information during the study that may affect your willingness to continue to participate, investigators will inform you promptly.

8. Whether you will participate in this study or not depends entirely on your own choice. You may refuse to participate in the study or withdraw from the study at any time during the study without prejudice to your relationship with investigators or loss of medical or other benefits.

Investigator may suspend your participation in this study at any time in your best interest.

If you do not want to continue participating in the study, or if you drop out, there are many alternative treatments available. You do not have to choose to participate in the study to treat the disease.

If you withdraw from the study for any reason, you may be asked about your use of the study drugs. You may also be required to undergo laboratory tests and physical examination if investigators deem it is necessary.

If you choose to participate in this study, we expect you to follow through with the entire study.

9. It is up to you to decide whether to participate in the study or not. You may discuss with your family or friends. Before you decide to participate in the study, please consult investigators as many questions as possible until you fully understand the study.

Thank you for reading the above material. If you decide to participate in the study, please let investigators know and he/she will make all the arrangements for you.

Please keep this document.

Informed Consent • Consent Signature Page

Statement of Consent:

1. I have read the introduction of this study and had the opportunity to discuss with investigators and ask questions.

2. I know the benefits and possible risks of participating in this study. I acknowledge participation in the study is voluntary, and I'm sure there's plenty of time for me to consider and understand:

(1) I told the investigators my medical history in detail at the first visit, and took corresponding examinations and tests so that investigators could make an accurate judgment and decide whether to participate in this study or not.

(2) If I meet the requirements, I will receive systematic treatment; And during the treatment, I need to take corresponding examinations and tests; If I need to take other medications due to changes in my condition, I will ask investigators for advice in advance, or tell investigators truthfully afterwards.

(3) I also know that if I quit the study during the study, especially if I quit the study due to drug reasons, I need to tell investigators about the change of my physical condition and complete the corresponding examinations, which will be very beneficial to myself and the whole study.

(4) I have access to information related to the study at any time, and I have the right to withdraw from the study at any time without discrimination or retaliation at any stage of the study. My withdrawal from the study will not affect my acceptance of other effective treatments.

(5) I consent to have access to my research materials by the drug regulatory authority, ethics committee or sponsor delegation.

(6) I consent to the use of my medical records in studies other than this one.

I have understood the above situation and have decided to participate in this clinical study with my written consent.

Participant (or agent) signature：______________

Telephone number：______________

Signature date：______________

I confirm that the details of this study, including their rights, benefits, and possible risks, have been explained to participants.

Investigator signature：______________

Telephone number：______________

Signature date：______________
